# Autoantibodies to Tumor-Associated Antigens in Epithelial Ovarian Carcinoma

**DOI:** 10.1155/2009/581939

**Published:** 2010-01-17

**Authors:** Benjamin Piura, Ettie Piura

**Affiliations:** ^1^Unit of Gynecologic Oncology, Department of Obstetrics and Gynecology, Soroka Medical Center and Faculty of Health Sciences, Ben Gurion University of the Negev, Beer Sheva 84101, Israel; ^2^Department of Obstetrics and Gynecology, Sapir Medical Center, Sackler School of Medicine, University of Tel-Aviv, Kfar Saba 44281, Israel

## Abstract

This review will focus on recent knowledge related to circulating autoantibodies (AAbs) to tumor-associated antigens (TAAs) in epithelial ovarian carcinoma. So far, the following TAAs have been identified to elicit circulating AAbs in epithelial ovarian carcinoma: p53, homeobox proteins (HOXA7, HOXB7), heat shock proteins (HSP-27, HSP-90), cathepsin D, cancer-testis antigens (NY-ESO-1/LAGE-1), MUC1, GIPC-1, IL-8, Ep-CAM, and S100A7. Since AAbs to TAAs have been identified in the circulation of patients with early-stage cancer, it has been speculated that the assessment of a panel of AAbs specific for epithelial ovarian carcinoma TAAs might hold great potential as a novel tool for early diagnosis of epithelial ovarian carcinoma.

## 1. Introduction

The development of circulating autoantibodies (AAbs) to tumor-associated antigens (TAAs) has been observed to be associated with cancer [1, 2]. Unlike traditional tumor markers (e.g., CA-125, CA-15-3, CA-19-9, and CEA), which are soluble proteins shed by bulky tumors, circulating AAbs to TAAs are detectable even when the tumor is very small and TAA expression is minimal [2]. Thus, the identification of AAbs to TAAs could potentially be used as a novel tool for early diagnosis of cancer [2–6]. Sahin et al. [7] introduced in 1995 an approach that has broad applicability to the analysis of the humoral immune response to cancer. This method, called SEREX (serological analysis of recombinant cDNA expression libraries), involves the immunoscreening of cDNA libraries prepared from tumor specimens with autologous sera. So far, over 2,000 candidate TAAs in many types of human cancer have been identified and separated into six categories [5, 8–11]: (1) differentiation antigens (expressed by cancers and a restricted subset of normal cells, e.g., tyrosinase, melan-A/MART-1, NY-BR-1, and gp100), (2) mutational antigens (e.g., CDK4, *β*-catenin, caspase-8, and p53), (3) amplification (overexpression) antigens (e.g., Her2/neu, NY-C0-58, and p53), (4) splice variant antigens (e.g., NY-CO-37/PDZ-45, and ING1), (5) viral antigens (e.g., HPV and EBV), and (6) cancer-testis (CT) antigens (e.g., MAGE, NY-ESO-1, and LAGE-1). The humoral immune response elicited by TAAs could have two major clinical applications [9]: (1) AAbs to TAAs could represent novel biomarkers for cancer diagnosis, prognosis, monitoring, and prediction of response to chemotherapy, (2) TAAs might be used as targets for immunotherapy of cancer. Notwithstanding, efforts to predict cancer based on autoimmunity to either an individual TAA or even tailor-made panel of TAAs have not yet resulted in serologic biomarkers with definitive predicting specificity and sensitivity [12]. It has, however, been shown by some investigators that the use of tailor-made panel of TAAs, rather than individual TAAs, enhances the likelihood of detecting cancer-associated AAbs with potential diagnostic value [3, 12].

Epithelial ovarian carcinoma is diagnosed in approximately 25,000 women yearly and causes approximately 16,000 deaths each year in the USA, with a worldwide incidence and mortality of approximately 200,000 and 115,000, respectively. It is the leading cause of death from gynecological malignancies in the USA and is the second leading cause of death, after uterine cervix cancer, due to gynecological malignancies in the world [6, 11, 13, 14]. Despite modest improvements in response rate, progression-free interval, and median survival due to multimodality treatment including cytoreductive surgery and chemotherapy comprised of platinum compound and paclitaxel, overall survival rates for epithelial ovarian carcinoma patients remain disappointing [11]. This has mainly been attributed to lack of early diagnosis and ineffective treatments. More than 75% of patients diagnosed with epithelial ovarian carcinoma have an advanced disease (FIGO stage III/IV), which has a five-year survival rate of less than 30% [2, 6, 9]. Although most patients initially respond to chemotherapy, including complete responses, the relapse rate is ~85%. Within two years of cytoreductive surgery and chemotherapy, tumors usually recur, and once relapse occurs, there is no known curative therapy. The use of serum-soluble tumor antigens, such as CA-125 glycoprotein, as biomarkers for detection of epithelial ovarian carcinoma has been limited by their insufficient specificity and sensitivity, particularly for organ-confined early-stage disease [6, 15]. Elevated levels of CA-125, the most widely used serum biomarker for epithelial ovarian carcinoma, occur in only 50% of stage I patients and can also be detected in healthy women [6, 15].

Recently, a model of surface epithelial ovarian tumors that includes two main pathways of tumorigenesis corresponding to type I and type II tumors has been proposed [16–19]. Type I tumors account for 25% of epithelial ovarian tumors and are made up of low-grade serous carcinomas, low-grade endometrioid carcinomas, most clear cell carcinomas, mucinous carcinomas, and malignant Brenner tumors. They develop in a stepwise fashion from well-recognized precursors, namely, borderline tumors which in turn develop from benign cystadenomas or adenofibromas. The benign tumors appear to develop from the ovarian surface epithelium (OSE) or cortical inclusion cysts (CIC) in the case of serous and mucinous tumors and from endometriosis or endometriomas in the case of endometrioid and clear cell tumors. Most Type I tumors are slow growing as evidenced by the observation that they are generally large and are often confined to the ovary at diagnosis. Type II carcinomas account for 75% of epithelial ovarian tumors and include high-grade (“moderately” and “poorly” differentiated) serous carcinomas, high-grade endometrioid carcinomas, few clear cell carcinomas, undifferentiated carcinomas, and carcinosarcomas. Type II carcinomas evolve rapidly, disseminate early in their course, and are highly aggressive. As these tumors are rarely associated with morphologically recognizable precursor lesions, it has been proposed that they develop “de novo” from the OSE or CIC [16–19]. Although type II carcinomas are rarely associated with morphologically recognizable precursor lesions, it has been recently suggested that precursor lesions of type II carcinomas may arise from dysplasia in inclusion cysts or serous intraepithelial carcinoma in the fallopian tubes. These precursor lesions may be difficult to recognize because they presumably undergo rapid transit from an occult lesion to a clinically diagnosed overt high-grade carcinoma [17]. Thus, there is a need to discover novel biomarkers, such as AAbs to specific ovarian carcinoma TAAs, for early diagnosis, prediction of prognosis, and monitoring of treatment, and to develop new therapeutic approaches, such as immunotherapy, for the management of this disease [2, 6, 15].

The establishment of AAbs to TAAs as biomarkers for epithelial ovarian carcinoma and the development of successful immunotherapeutic strategies require the identification and characterization of immunogenic epithelial ovarian carcinoma TAAs that will be recognized by the host immune system [2, 15]. Barua et al. [6] demonstrated with use of an enzyme-linked immunosorbent assay (ELISA) that epithelial ovarian carcinoma patients have significantly higher levels of serum AAbs to proteins extracted from normal ovarian tissue (*P* < .001) and proteins extracted from ovarian carcinoma tissue (*P* < .001) compared to those of healthy women. The proportion of sera positive for AAbs to normal ovarian tissue (81%, *P* < .001) and ovarian carcinoma tissue (69%, *P* < .001) was significantly higher in epithelial ovarian carcinoma patients compared to that of healthy controls [6]. In epithelial ovarian carcinoma patients, there was no significant difference in AAbs detection using antigens from normal ovary or from ovarian carcinoma, and in addition, there was no difference in AAbs prevalence by disease stage [6]. Based on these results, the authors concluded that AAbs to TAAs are a potential useful diagnostic biomarker for epithelial ovarian carcinoma [6]. Nevertheless, only few circulating AAbs to specific epithelial ovarian carcinoma TAAs have been identified and investigated so far [2, 6, 9, 15, 20]. In epithelial ovarian carcinoma, like in other malignancies, the use of tailor-made panel of TAAs, rather than individual TAAs, enhances the likelihood of detecting cancer-associated AAbs with potential diagnostic value. By the application of Bayesian modeling of autologous antibody responses against ovarian TAAs, Erkanli et al. [21] demonstrated that measuring specific AAbs to a three-member panel of TAAs (p53, NY-CO-8, and HOXB7), in addition to serum CA-125, yielded a reasonable sensitivity and specificity in discriminating between epithelial ovarian carcinoma patients and healthy controls.

This paper will review the up-to-date knowledge related to AAbs to TAAs in epithelial ovarian carcinoma. Table 1 shows the frequency of identified AAbs to epithelial ovarian carcinoma TAAs.

## 2. Autoantibodies to p53 Protein

The wild-type p53 gene is a tumor suppressor gene located on chromosome 17p13 and encodes a 53-kDa nuclear phosphoprotein that normally acts as a guardian of the integrity of the genome and, thus, has been called “guardian of the genome” [22–25]. p53 gene aberrations are the most common genetic changes found in human malignancies [22, 23, 26, 27]. Missense point mutations, which represent more than 85% of gene abnormalities, lead to a conformational change which stabilizes the p53 protein and allows it to accumulate in the nucleus to relatively high levels [23, 24, 26, 28, 29]. Accumulation of the mutant p53 in tumor cells can elicit a humoral immune response leading to the production of anti-p53 AAbs [22, 23]. In fact, serum anti-p53 AAbs were found in 3.5% to 30% of patients with different malignancies and, in particular, in 15% to 29% of women with ovarian tumor [22–24, 30–32]. Indeed, while mutation of p53 appears a seminal event in carcinogenesis and is present in 80% of type II epithelial ovarian carcinoma, it is still unclear why only a subset (20%–40%) of these cases generates anti-p53 AAbs [33]. At first, it was thought that only tumors with missense p53 mutations resulting in p53 overexpression can elicit anti-p53 AAbs [22, 34–36]. Anti-p53 AAbs have, however, also been detected in sera from patients with tumors lacking p53 overexpression, and induction of anti-p53 AAbs in these patients might be due to the unusual presentation of large amounts of wild-type p53 from necrotic large tumors or metastases [22, 37]. In recent years, it has been shown that anti-p53 AAbs are directed against immunodominant epitopes localized in the amino and carboxy terminal ends of the p53 protein, unrelated to the mutational hot spot [22, 28, 38–40]. Some investigators have found that tumors bearing p53 aberrations are less sensitive to cisplatin and doxorubicin exposure, and cisplatin represents the most active compound of standard chemotherapy regimens in the treatment of epithelial ovarian carcinoma [24, 41, 42]. Because circulating anti-p53 AAbs occur only rarely in individuals with nonmalignant diseases, it is tempting to speculate that these AAbs are reliable indicators of malignancy [28]. Ovarian carcinoma is regarded as a tumor entity associated with the highest prevalence and titers of circulating anti-p53 AAbs [24, 28, 31]. In other words, ovarian carcinomas are among the most immunogenic malignancies inducing anti-p53 AAbs response [28].

Gadducci et al. [43] demonstrated by ELISA that preoperative serum anti-p53 AAbs were present in 33% of 30 ovarian carcinoma patients. Angelopoulou et al. [30] showed with use of a time-resolved immunofluorometric technique that anti-p53 AAbs were present in 24% of 174 ovarian carcinoma patients. The presence of anti-p53 AAbs was related to tumor grade (G1, 5.9%; G2, 38.5%; G3, 27.8%; *P* = .001) but not to stage. Univariate analysis showed that ovarian carcinoma patients with serum anti-p53 AAbs had significantly shorter disease-free survival than those without anti-p53 AAbs (*P* = .02) whereas overall survival was not significantly different. Multivariate analysis, however, demonstrated that the presence of anti-p53 AAbs was not an independent prognostic factor for disease-free survival or overall survival [30]. In another study, Gadducci et al. [22] investigated with use of a new generation ELISA the presence of anti-p53 AAbs in blood samples preoperatively drawn from 86 women with ovarian carcinoma. Serum anti-p53 AAbs were found in 3 (10.0%) of the 30 women with stage I/II disease and 15 (26.8%) of 56 women with stage III/IV disease (*P* = .09). Forty-four patients with stage III/IV disease who had six cycles of adjuvant platinum-based chemotherapy were assessed in detail. A pathological complete response at second-look surgery was achieved by none (0%) of the 15 patients with serum anti-p53 AAbs compared to 7 (24.1%) of the 29 patients without anti-p53 AAbs (*P* = .09). The preoperative serum anti-p53 antibody status had no prognostic relevance for overall survival and progression-free survival. It was concluded that the assessment of preoperative serum anti-p53 AAbs seems to have a limited clinical value in the management of women with advanced-stage epithelial ovarian carcinoma [22]. Soussi [23] surveyed literature from 1979 through 1999 on anti-p53 AAbs in the sera of patients with various types of cancer and found 11 studies that examined anti-p53 AAbs in the sera of ovarian carcinoma patients. The frequency of anti-p53 AAbs in the sera of ovarian carcinoma patients ranged in the various studies from 8.7% to 92.3%. Overall, the presence of anti-p53 AAbs was demonstrated in the sera of 143/652 (21.9%) ovarian carcinoma patients. With use of x^2^ test, it has been shown that the frequency of anti-p53 AAbs was significantly higher in the sera of epithelial ovarian carcinoma patients compared to that of healthy individuals (*P* < 10^−4^) [23]. Only few studies demonstrated that the presence of anti-p53 AAbs in the sera of ovarian carcinoma patients was associated with a poor histological differentiation [32] and poor survival [30, 44]. Vogl et al. [38] reported in 1999 that anti-p53 AAbs were identified with use of an ELISA in the sera of 38/83 (46%) ovarian carcinoma patients. Anti-p53 AAbs were detectable at all stages of disease and were more frequent in patients with higher age (*P* = .014), postmenopausal status (*P* = .050), and advanced-stage disease (*P* = .046) [38]. Multivariate analysis demonstrated that patients with anti-p53 AAbs had a 1.96-fold risk for relapse (95% confidence interval, 1.02–3.78) [38]. In 2000, Vogl et al. [28] analyzed with use of a newly developed ELISA based on highly purified and re-natured p53 the presence of anti-p53 AAbs in the sera of 113 patients with ovarian carcinoma, 15 patients with borderline ovarian tumor, and 117 patients with benign ovarian tumor. The prevalence of anti-p53 AAbs in patients with invasive cancer was 19% (21/113), whereas no anti-p53 AAbs were found in patients with ovarian borderline or benign tumors [28]. Anti-p53 AAbs were only detectable in patients with immunohistochemical staining of nuclear p53 in the tumor (*P* = .006). Presence of anti-p53 AAb positively correlated with tumor stage (*P* = .034) and grade (*P* = .009). Kaplan-Meier analysis showed both a shortened overall survival (*P* = .0016) and relapse-free survival (*P* = .055) for anti-p53 AAb-positive patients. High titers of anti-p53 AAbs related to even worse prognosis. Anti-p53 AAbs independently related to poor survival adjusting for stage (*P* = .026), grade (*P* = .029), and residual disease after surgery (*P* = .005). The authors concluded that preoperative findings of adnexal mass with serum anti-p53 AAbs are strongly suggestive of an aggressive invasive ovarian cancer [28]. Abendstein et al. [24] evaluated the prognostic significance of preoperative serum and ascitic anti-p53 AAbs in advanced-stage ovarian carcinoma. In 113 patients with significant amounts of ascites, serum and ascitic anti-p53 AAbs were detected in 28 (24.8%) and 21 (18.6%) patients, respectively. Univariate analysis showed that detection of anti-p53 antibodies in ascites, but not in the serum, is a significant predictor of adverse disease-free (*P* = .003) and overall survival (*P* = .01). Multivariate analysis, however, demonstrated that ascitic anti-p53 positivity is a significant predictor of only adverse progression-free survival (*P* = .01) [24].

With the use of an ELISA, Hogdall et al. [45] demonstrated preoperative serum IgG anti-p53 AAbs in 24/193 (13%) ovarian carcinoma patients, 0/34 (0%) patients with ovarian borderline tumors, and 0/86 (0%) healthy controls. No significant associations were demonstrated between anti-p53 AAbs and clinical stage, age, histological subtype, and extensiveness of primary surgery. Significantly elevated serum CA-125 levels in anti-p53 AAb-positive patients compared to lower serum CA-125 levels in anti-p53 AAb-negative patients (*P* = .003) were observed. In univariate and multivariate survival analyses, however, no significant differences were demonstrated between anti-p53 AAb-positive and anti-p53 AAb-negative ovarian carcinoma patients [45]. Since a low sensitivity for anti-p53 AAbs alone and no major additional effect of serum CA-125 level were demonstrated, it was concluded that serum anti-p53 AAbs levels are of no diagnostic value, even if combined with the tumor marker CA-125 [45].

In contrast to the rarity of p53 mutations in type I tumors, p53 mutations are identified in 50%–80% of advanced-stage (stage III and IV) type II epithelial ovarian carcinomas and in approximately 40% of early-stage (stage I and II) type II epithelial ovarian carcinomas [16, 18]. This has led to the suggestion that p53 mutation is an early seminal event in the development of type II epithelial ovarian carcinomas [17]. Moreover, in patients carrying BRCA1 or BRCA2 germline mutations and, as a result of this, are at higher risk for ovarian carcinoma, p53 mutations are found at high frequency, even in very early-stage I high-grade serous carcinoma identified upon prophylactic bilateral salpingo-oophorectomy [33]. Overexpression and mutation of p53 were observed not only in these microscopic invasive carcinomas but also in adjacent dysplastic epithelium. Early serous carcinomas were observed to occur predominantly in the fallopian tube fimbriae of women with BRCA germline mutations. Recently, a “p53 signature,” described as normal tissue morphology but p53 immunostaining positive (possibly associated with p53 mutation) in the secretory epithelial cells of the fallopian tube fimbriae, has been proposed as the precursor of type II carcinomas [14, 46, 47]. The p53 signature is also associated with DNA damage, as observed using *γ*-H2AX immunostaining as a biomarker. Tubal intraepithelial carcinomas are not considered precursor lesions but rather type II frank malignancies that will eventually spread if they remain undetected [33]. The common occurrence of p53 signatures in women with and without BRCA mutations suggests that it is independent of BRCA status. However, women at high genetic risk may be more likely to progress from a p53 signature to an intraepithelial or invasive carcinoma. It is interesting to note that women with mutations in BRCA have up to a 46% lifetime risk of ovarian cancer and that the prevalence of p53 signatures is 38% in this population, suggesting that in fact the rate of progression is high [14]. In other words, the high frequency of p53 signatures in fallopian tubes from all women, combined with the much higher risk of cancer in BRCA+ women, suggests that a germline BRCA mutation may serve as a promoter, enhancing the risk of transition from a p53 signature to malignancy [46]. Obviously, tests that detect mutant p53 or autoantibodies to mutant p53 protein in the blood could prove to be useful for detecting early low-volume type II epithelial ovarian carcinoma [17, 18].

Tsai-Turton et al. [33] demonstrated that serum anti-p53 AAb levels were significantly higher in patients with type II ovarian carcinoma as compared to those of healthy women (*P* < .001). At a cutoff level of 5.4 U/mL, anti-p53 AAb seropositivity was detected in 25% of the 116 type II ovarian carcinoma patients, whereas no anti-p53 AAb seropositivity was seen among the 14 women with type I ovarian tumor, 20 with atypical proliferative tumors, and 39 with benign ovarian cysts (*P* < .0001) [33]. A significantly higher serum anti-p53 AAb level was found in patients with advanced-stage (III/IV) type II carcinoma as compared to patients with early-stage (I/II) type II carcinoma (*P* < .001). Type II carcinoma patients whose tumor contained any p53 mutation exhibited significantly higher serum anti-p53 AAb levels than those whose tumor contained only wild-type p53 (*P* = .0019). There was no obvious association between mutation in a particular exon and serum anti-p53 AAb levels [33]. No significant correlation was observed between serum CA-125 levels and serum anti-p53 AAb levels (*P* = .8). Of 17 cytokines tested (IL-1*β*, IL2, IL4, IL5, IL6, IL7, IL8, IL10, IL12, IL13, IL17, G-CSF, GM-CSF, IFN-*γ*, MCP-1 (MCAF), MIP-1*β*, and TNF-*α*), only the serum levels of cytokines IL5, IL6, IL-8, IL10, MCP-1, GM-CSF and TNF-*α* were significantly increased in type II carcinoma patients as compared to those in healthy women. The serum levels of IL6, IL-8, and IL10 were also significantly elevated in type I ovarian tumor as compared to those of healthy women. No significant differences were noted in the plasma levels of each of the 17 cytokines or CA-125 in ovarian carcinoma patients with wild type p53 versus mutated p53, with the exception of IL6 (*P* = .02) and IL10 (*P* = .007) that were significantly higher in the sera of those with mutated p53. An examination of correlation was performed between the serum levels of each of the 17 cytokines and serum anti-p53 AAbs in patients with advanced-stage type II carcinoma. Only the absolute levels of IL4 (*P* = .019) and IL12 (*P* = .024) exhibited a significant correlation with the levels of anti-p53 AAbs. However, upon comparison of cytokine levels and anti-p53 AAb seropositivity at a cutoff level of 5.4 U/mL, there was no correlation with the levels of any cytokine, with the exception of IL-8. Women with advanced-stage type II carcinoma who were also anti-p53 AAb seropositive exhibited lower serum levels of IL-8 than those of seronegative women (*P* = .002) [33]. No association between anti-p53 AAb seropositivity and survival was observed (*P* = .29) among type II carcinoma patients. Likewise, there was no correlation between any of the 17 cytokine levels tested and the survival of patients with advanced-stage type II carcinoma. Tsai-Turton et al.'s conclusions are that [33] (1) type II, but not type I, ovarian carcinoma patients have elevated serum anti-p53 AAb levels and (2) anti-p53 AAbs are associated with mutation of p53, higher plasma IL4, and IL12 but lower plasma IL-8 levels and no survival advantage.

## 3. Autoantibodies to Homeobox Proteins

Homeobox genes are “master control genes” that act at the top of genetic hierarchies regulating cell growth, differentiation, and development [56–58]. Approximately 170 different vertebrate homeobox genes have been identified, of which 39 belong to the HOX family [56, 57]. Although it is well established that HOX proteins function as transcription factors, the vast majority of their targets have yet to be elucidated. It has been reported that HOXA5 regulates p53 transcription [59] and that p21 is a target of HOXA10 [60]. Expression of cell adhesion molecules is regulated by several HOX proteins [61]. Auto- and cross-regulatory interactions within the homeotic network have also been described [61]. Aberrant HOX gene expression observed in various malignancies has implicated their involvement in neoplasia [56, 57, 61]. The homeobox transcription factor genes HOXA7, HOXA9, HOXA10, and HOXA11 have an important role in the morphologic heterogeneity of epithelial ovarian cancers and their assumption of mullerian-like features [58]. Crijns et al. [62] demonstrated that MEIS and PBX homeobox proteins are extensively expressed in ovarian carcinomas and may play a role in ovarian carcinogenesis. Widschwendter et al. [63] demonstrated that DNA methylation of HOXA9 and HOXA11 genes in normal endometrium was associated with ovarian cancer. The overall risk of ovarian cancer was increased 12.3 folds by high HOXA9 methylation for all stages and 14.8 folds for early-stage ovarian cancers, independent of age, phase of the menstrual cycle, and histology of the cancer [63]. It has been shown that overexpressed and aberrant products of the homeobox genes in cancer patients can elicit an autologous immune response.

In contrast to very little or no expression of HOXA7 protein in normal ovarian surface epithelium (OSE), increased expression of HOXA7 protein was demonstrated in columnar cells lining invaginations of the OSE, in mullerian-like epithelium of inclusion cysts of the OSE and in normal epithelium of the fallopian tube [15]. These observations raise the possibility that HOXA7 expression is associated with normal development of the mullerian duct-derived epithelia and that inappropriate expression of HOXA7 in the OSE could give rise to aberrant epithelial differentiation leading to the development of epithelial ovarian carcinoma. Thus, anti-HOXA7 AAbs represent potential serum biomarkers, particularly for detecting small, early-stage ovarian carcinomas [15]. With use of an ELISA employing purified recombinant HOXA7 protein, Naora et al. [15] detected anti-HOXA7 AAbs in the sera of 1/24 (4.1%) patients with poorly differentiated ovarian serous carcinoma, 16/24 (66.6%) patients with well-to moderately differentiated ovarian serous carcinoma, 13/19 (68.4%) patients with benign ovarian serous cystadenoma, and 0/30 (0%) healthy women. These findings indicate that an obvious deficiency in the utility of serum anti-HOXA7 AAbs as a diagnostic biomarker is its extremely poor sensitivity for detecting poorly differentiated ovarian carcinomas [15]. Nevertheless, although the anti-HOXA7 AAb assay alone allows no distinction between benign and malignant ovarian tumors, it is able to discriminate patients with well-to moderately differentiated ovarian carcinomas from healthy women [15].

Ovarian carcinomas were found to express HOXB7, a product of a homeobox gene, at markedly higher levels than normal OSE [21, 48]. Overexpression of HOXB7 in immortalized normal OSE cells upregulated expression of basic fibroblast growth factor (bFGF), a potent mitogenic and angiogenic factor and dramatically increased OSE cell proliferation [21, 48]. This indicates that HOXB7 overexpression could play a significant role in growth of ovarian carcinomas. Naora et al. [48] revealed significant serologic reactivity to the HOXB7 antigen in 13/39 (33.3%) of ovarian carcinoma patients and in only 1/29 (3.4%) of healthy women (*P* < .0001). This preliminary observation needs to be validated in larger case-control studies with particular attention being drawn to correlating titers of anti-HOXB7 AAbs with stage of disease. Nevertheless, Naora et al.'s data raise the possibility that serologic detection of anti-HOXB7 AAbs could have diagnostic potential [48].

## 4. Autoantibodies to Heat Shock Proteins

By ELISA using a purified recombinant 27-kd heat shock protein (HSP-27), Korneeva et al. [49] demonstrated a significantly higher prevalence of serum IgG AAbs to HSP-27 in patients with ovarian carcinoma (17/34, 50%) and other female genital tract malignancies compared to those in healthy women (3.4%). AAbs to other heat shock proteins (HSP-60, HSP-70, and HSP-90) were also detected in some patients with female genital tract malignancies, including ovarian carcinoma, but their prevalence was not different from that found in healthy controls [49]. Thus, HSP-27 appears to be uniquely autoantigenic after development of cancer of the female genital tract and may therefore be a marker for the presence of cancer of the female genital tract. Since the presence of AAbs to HSP-27 has been associated with an improved survival among patients with breast cancer, it has been speculated that these AAbs may directly aid in the immune defense against cancer [49]. Another possibility, as suggested by Conroy et al. [64], is that autoimmunity to HSP-27 is not directly associated with survival. Instead, HSP-27 may be a surrogate marker for immunity to a TAA that is bound to this heat shock protein [64]. Further studies on autoimmunity to HSP-27 and characterization of HSP-27-TAA complexes could lead to new methodologies to boost immune defenses against cancer [64].

With use of SEREX, Luo et al. [9] identified 12 new candidate ovarian carcinoma antigens. One of them, heat shock protein 90 (HSP-90), was studied in detail and found to be immunogenic in one-third of the patients with advanced-stage ovarian carcinoma. Anti-HSP-90 AAbs were detected in the sera of 7/22 (32%) patients with stage III/IV ovarian carcinoma, 1/10 (10%) patients with stage I/II ovarian carcinoma, 1/37 (2.7%) colorectal carcinoma patients, 1/13 (7.7%) breast carcinoma patients, and 1/20 (5%) patients with benign gynecologic disease [9]. Based on the finding that anti-HSP-90 AAbs are frequently found in advanced-stage epithelial ovarian carcinoma, it has been concluded that anti-HSP-90 AAbs might represent a novel biomarker for epithelial ovarian carcinoma and that HSP-90 might be used as a target for immunotherapy in this disease [9].

## 5. Autoantibodies to Cathepsin D

Primary biological function of enzymatically active cathepsin D is protein degradation in an acidic milieu of lysosomes [65]. Failure of this function resulted in accumulation of lipofuscin in variety of cell types, neurodegeneration, developmental regression, and visual loss. Procathepsin D, secreted from cancer cells, acts as a mitogen on both cancer and stromal cells and stimulates their proinvasive and prometastatic properties. Procathepsin D/cathepsin D levels represent an independent prognostic factor in a variety of cancers [65].

Both 52-kDa procathepsin D and 32-kDa mature cathepsin D were shown to elicit a humoral immune response in epithelial ovarian carcinoma patients [50, 66]. No significant difference was detected in the immunoreactivity of patient serum with the glycosylated and deglycosylated forms of the cathepsin D, suggesting that patient humoral responses are directed primarily against the core protein [50, 66]. Four immunogenic epitopes of cathepsin D were identified: two peptides within the procathepsin D, the third at the carboxy terminus, and the fourth at the glycosylation site of the mature enzyme [50, 66]. The identification of specific antigenic epitopes of cathepsin D may be useful in defining effective targets for directed active immunotherapy for epithelial ovarian carcinoma. Three mechanisms leading to the generation of AAbs to cathepsin D in epithelial ovarian carcinoma patients have been identified: overexpression of cathepsin D resulting from amplification or increased protein stability, altered glycosylation of cathepsin D at amino acid 134, and inappropriate extracellular release of cathepsin D [50, 66]. Chinni et al. [50] found that 10/25 (40%) epithelial ovarian carcinoma patients produced circulating AAbs to cathepsin D. It has been concluded that identifying TAAs that elicit humoral immune response and defining antigenic epitopes may provide new avenues for depicting antigenic targets for directed immunotherapy [50, 66]. Recently, Taylor et al. [67] showed that the presence of AAbs against cathepsin D can differentiate between benign ovarian masses and ovarian carcinomas (even stage I carcinoma).

## 6. Autoantibodies to Cancer-Testis Antigens

The cancer-testis (CT) antigens are a distinct and unique class of differentiation antigens that are expressed in a variety of cancers, but not in normal adult tissues, except for germ cells of the testis, and hence appear to be ideal targets for immunotherapy [10, 11, 68]. The NY-ESO-1 or LAGE-1 antigens belong to the group of CT antigens. The NY-ESO-1 gene is located on Xq28 [9]. Stockert et al. [51] reported an AAb response to NY-ESO-1/LAGE-1 antigen in 12.5% of epithelial ovarian carcinoma patients. Odunsi et al. [11] showed that serum AAbs to CT antigens NY-ESO-1 and LAGE-1 were detected in 11/37 (30%) epithelial ovarian carcinoma patients whose tumors expressed either NY-ESO-1 or LAGE-1 antigens. Detectable AAbs were present for up to 3 years after initial diagnosis of epithelial ovarian carcinoma. Although there was no statistically significant association between NY-ESO-1/LAGE-1 antigens expression and patients' survival, the data showed aberrant expression of NY-ESO-1 and LAGE-1 in a significant proportion of epithelial ovarian carcinoma patients [11]. These findings indicate that AAbs to NY-ESO-1 and LAGE-1 antigens might serve as a biomarker of epithelial ovarian carcinoma and that NY-ESO-1 and LAGE-1 antigens are attractive targets for antigen-specific immunotherapy in epithelial ovarian carcinoma [11].

## 7. Autoantibodies to MUC1 Protein

Polymorphic epithelial mucin (PEM, MUC1), a human mucin family member, is a high-molecular-weight (over 400 kDa) transmembrane glycoprotein. It is expressed in a hyperglycosylated form and low levels by many types of normal epithelial cells and in a hypoglycosylated form and high levels by most epithelial adenocarcinomas including ovarian and breast carcinomas [52, 69]. About one-third of ovarian and breast carcinoma patients have circulating AAbs to MUC1, either free or bound to immune complexes. While the presence of these immune complexes has prognostic significance in cancer patients, the significance of free AAbs to MUC1 is less clear [70]. AAbs to MUC1 have been described and correlated with a more favorable prognosis; thus, it seems that risk for epithelial ovarian carcinoma might be reduced by preexisting MUC1-specific immunity [71, 72]. With use of an ELISA, Cramer et al. [52] investigated the presence of anti-MUC1 AAbs in the sera of 668 epithelial ovarian carcinoma patients. By a cutoff of optical density (OD) ≥ 0.6, 33.8% of controls and 45.8% of epithelial ovarian carcinoma patients were positive for anti-MUC1 AAbs. By a cutoff of OD ≥ 1.0, 12.3% of controls and 25% of ovarian carcinoma patients had a high level of anti-MUC1 AAbs. The authors found that factors known to decrease the risk of developing epithelial ovarian carcinoma, such as oral contraceptive use and nonuse of talc in genital hygiene, are associated with presence of circulating anti-MUC1 AAbs [52]. This supports the notion that preexisting AAbs to MUC1 may reduce the risk of developing epithelial ovarian carcinoma, and presence of AAbs to MUC1 in epithelial ovarian carcinoma is correlated with a more favorable prognosis [52].

## 8. Autoantibodies to GIPC-1 Protein

The protein known as GIPC-1, a member of a family of PDZ-domain conserved proteins, is involved in regulation of G-protein signaling and is upregulated in ovarian and breast carcinomas [53, 73–75]. With use of 27.B1 and 27.F7 human monoclonal antibody specific to GIPC-1 protein, Yavelsky et al. [53] demonstrated, respectively, a positive immunnohistochemical staining for GIPC-1 in 7/13 (54%) and 8/15 (53%) of ovarian serous carcinomas, 4/11 (36%) and 0/11 (0%) of ovarian borderline serous tumors, 2/15 (14%) and 3/15 (21%) of ovarian serous cystadenomas, and 0/8 (0%) and 0/8 (0%) of normal ovarian tissues. Based on these results, the authors [53] hypothesize that detection of serum AAbs to GIPC-1 might be a sensitive marker for early-stage epithelial ovarian carcinoma and superior to methodologies based on TAA detection. With use of a novel technique of chemiluminescent optical fiber immunoassay (the instrument is called chemiluminescent optical fiber immunosensor), Salama et al. [75] tested sera from 11 epithelial ovarian carcinoma patients, 22 breast carcinoma patients, and asymptomatic controls for the presence of IgM anti-GIPC-1 AAbs. The chemiluminescent optical fiber immunosensor detected 54% and 77% anti-GIPC-1 AAbs-positive sera within ovarian and breast carcinoma patients, respectively, as compared to ELISA, which only detected 18% and 27%, respectively [75]. The authors conclude that the chemiluminescent optical fiber immunoassay is an efficient technique for prompt detection of AAbs to TAAs and, thus, foresee that the newly developed chemiluminescent optical fiber immunosensor might serve as an efficient tool for early diagnosis of ovarian and breast carcinomas [75].

## 9. Autoantibodies to IL-8

IL-8 belongs to the superfamily of CXC chemokines attracting neutrophils and macrophages and manifests a wide range of proinflammatory effects. In addition, IL-8 was found to be a potent proangiogenic factor and is able to promote tumor cell proliferation, modulate collagenase production, and affect metastasis formation. IL-8 is produced by various malignant cells, and increased blood levels of IL-8 have been demonstrated in several malignancies including ovarian cancer [54]. Recently, Tsai-Turton et al. [33] showed that women with advanced-stage type II ovarian carcinoma who were also anti-p53 AAb seropositive exhibited lower serum levels of IL-8 than seronegative cases (*P* = .002). With use of an immunofluorescent bead-based assay, Lokshin et al. [54] measured circulating IL-8 and anti-IL-8 IgG AAbs in sera from 44 patients with early-stage (I-II) epithelial ovarian carcinoma, 50 patients with advanced-stage (III-IV) epithelial ovarian carcinoma, 37 patients with benign pelvic masses, and 80 healthy women. Concentrations of serum anti-IL-8 IgG AAbs in epithelial ovarian carcinoma patients varied from 2.8 to 250.7 ng/mL. Mean concentrations of anti-IL-8 IgG AAbs were significantly higher in patients with both early-stage (I-II) (*P* < .01) and advanced-stage (III-IV) (*P* < .05) epithelial ovarian carcinomas as compared with healthy controls but were also elevated in the sera of patients with benign pelvic masses. Anti-IL-8 IgG AAbs concentrations did not differ significantly between patients with early (I-II) versus late (II–IV) stages of epithelial ovarian carcinoma. However, when concentrations of anti-IL-8 IgG AAbs were analyzed in a group of patients with only stage I epithelial ovarian carcinoma, the differences between cancer and control group did not reach statistically significant levels [54]. Logistic regression analysis of concentrations of anti-IL-8 IgG AAbs in patients with stages I and II epithelial ovarian carcinoma versus healthy controls allowed for prediction of early epithelial ovarian carcinoma with 98% specificity and 65.5% sensitivity. Combining IL-8 and anti-IL-8 IgG AAbs with CA-125 resulted in increased classification power as compared to individual markers analyzed separately. The authors concluded that IL-8 and anti-IL-8 AAbs might potentially serve as additional biomarkers for epithelial ovarian carcinoma [54].

## 10. Autoantibodies to Ep-CAM

Epithelial cell adhesion molecule (Ep-CAM) is a ubiquitous antigen in epithelial cells that is highly overexpressed in epithelial ovarian carcinoma as well as other cancers, and its expression is correlated with malignant proliferation [55]. With use of an ELISA, Kim et al. [55] examined AAbs to Ep-CAM in sera of 52 epithelial ovarian carcinoma patients, 26 patients with benign ovarian disease, and 26 normal controls. The mean optic density (OD) levels of AAbs to Ep-CAM in cancer patients, benign ovarian disease, and normal controls were 0.132, 0.098, and 0.090, respectively. The difference between cancer cases and the other cases was statistically significant (*P* < .05). The sera of stage IV epithelial ovarian carcinomas showed lower levels of AAbs to Ep-CAM, compared with either stage I or II epithelial ovarian carcinomas (*P* < .05). Based on OD cutoff value 0.140 for AAbs to Ep-CAM, which was defined as the mean OD value of the normal controls +2 standard deviations (SD), 22 epithelial ovarian carcinoma patients (42.3%) were positive, whereas none of the control (0%) and 2 benign ovarian disease (7.7%) cases were positive [55]. A receiver operating curve (ROC) was plotted to investigate the optimal OD cutoff value and to compare AAbs to Ep-CAM with CA-125. Based on OD cutoff value of 0.115 for AAbs to Ep-CAM, 38 (73.1%) epithelial ovarian carcinoma patients were positive, whereas 6 (23.1%) benign ovarian disease cases and 5 (19.2%) normal cases were positive [55]. Thus, an OD cutoff value of 0.115 enabled AAbs to Ep-CAM to detect epithelial ovarian carcinoma with 73.1% sensitivity and 80.8% specificity. In comparison, with the use of a cutoff value 35 units/mL, CA-125 detected epithelial ovarian carcinoma with 86.5% sensitivity and 88.5% specificity. By using both AAbs to Ep-CAM and CA-125, epithelial ovarian carcinoma was detected with 90.4% sensitivity and 92.3% specificity [55]. It was concluded that although AAbs to Ep-CAM are less sensitive and specific than CA-125, AAbs to Ep-CAM might be complementary to CA-125. By combining AAbs to Ep-CAM with CA-125, the specificity is increased as compared with CA-125 alone without lowering the sensitivity. This means that the false positive ratio can be decreased by way of combining AAbs to Ep-CAM with CA-125 in screening and early diagnosis of epithelial ovarian carcinoma [55].

## 11. Autoantibodies to S100A7

By using the technique of two-dimensional differential gel electrophoresis analysis of immunoprecipitated tumor antigens (2D-DITA), Gagnon et al. [2] identified significantly higher expression of S100A7 protein (11-kDa protein expressed by a wide variety of cells and is involved in the regulation of cell cycle progression and differentiation) in epithelial ovarian carcinoma tissue compared to that in normal ovary. In addition, they identified by an ELISA significantly elevated levels of AAb to S100A7 protein in the plasma of epithelial ovarian carcinoma patients compared with those of healthy controls. Since significantly higher levels of S100A7 expression were observed in both early- and advanced-stage epithelial ovarian carcinoma compared with those benign and normal ovarian tissues, the authors [2] suggest that (1) increased S100A7 expression may be an early event in ovarian pathogenesis and (2) AAbs to S100A7 may serve as a useful biomarker for early diagnosis of epithelial ovarian carcinoma.

## 12. Conclusion

Epithelial ovarian carcinoma is highly lethal because it is typically asymptomatic until well advanced. Traditional diagnostic tools for early diagnosis of epithelial ovarian carcinoma, that is, manual pelvic examination, imaging studies (US, CT, MRI, PET, etc.), and measurement of serum CA-125 levels, are crippled with insufficient sensitivity and specificity [2, 6, 68]. Thus, at present, there is little to offer for early diagnosis of epithelial ovarian carcinoma. AAbs to TAAs have been shown to be present in the circulation of people with various forms of solid tumor even before TAAs can be detected, and these AAbs can be measured up to 5 years before symptomatic disease [2, 68, 76, 77]. Thus, evidently, the human immune system recognizes the autologous TAAs as “nonself” and makes a humoral immune response very early in the disease process [2]. Although measurement of AAbs to a single TAA is possible, the low sensitivity and specificity renders single AAb measurements of little value for screening and early detection of cancer. There has been some proof, however, that combination of AAbs to various TAAs into a panel assay test might provide a reasonable level of sensitivity and specificity for the detection of cancer [2, 4, 6, 21, 76–78]. Thus, preliminary data support the idea of developing a serum assay evaluating the antibody response to a panel of TAAs for cancer diagnosis [1]. The implications of this would be that AAbs to TAAs would provide a simple blood test for early diagnosis of epithelial ovarian carcinoma [2, 6]. Nevertheless, it must be remembered that measurement of serum AAbs to TAAs for early diagnosis of epithelial ovarian carcinoma is still investigational and should be carried out along with traditional diagnostic studies.

##  Conflicts of Interest 

The authors declare no conflicts of interest.

## Figures and Tables

**Table 1:** Frequency of identified circulating AAbs to TAAs in epithelial ovarian carcinoma.

AAb to TAA	Positive	Total	%	Comment	Reference
p53	10	30	33		[43]
p53	42	174	24	Association with shorter disease-free survival.	[30]
p53	18	86	21	No prognostic relevance for survival.	[22]
p53	143	652	22	Summary of 11 studies. Frequency of AAbs: 8.7%–92.3%. Few studies showed association with poor histological differentiation and poor survival.	[23]
p53	38	83	46	Association with higher risk for relapse.	[38]
p53	21	113	19	Correlation with tumor stage and grade. Association with worse survival.	[28]
p53	28	113	25	Serum AAbs had no prognostic relevance for survival. Ascitic AAbs were associated with adverse survival.	[24]
p53	24	193	13	No diagnostic value, even if combined with CA-125. No prognostic relevance for survival.	[45]
p53	29	116	25	Detected exclusively in type II carcinoma.	[33]
HOXA7	17	48	36	Allows no distinction between benign and malignant ovarian tumors.	[15]
HOXB7	13	39	33	Significant difference in the frequency of AAbs between ovarian carcinoma patients and healthy controls. Promising diagnostic potential.	[48]
HSP-27	17	34	50	Promising diagnostic potential. Possible association with improved survival.	[49]
HSP-90	8	32	25	Association with advanced-stage disease. Promising diagnostic potential.	[9]
Cathepsin D	10	25	40	Promising diagnostic potential.	[50]
NY-ESO-1/LAGE-1	NR	NR	13		[51]
NY-ESO-1/LAGE-1	11	37	30	No prognostic relevance for survival. Promising diagnostic potential.	[11]
MUC1	306	668	46	Correlation with a more favorable prognosis.	[52]
GIPC-1	6	11	54	Promising diagnostic potential.	[53]
IL-8	NR	94	NR	Anti-IL-8 AAb levels were elevated in ovarian carcinoma patients as compared with those of healthy controls. Promising diagnostic potential.	[54]
Ep-CAM	22	52	42	Potential diagnostic value.	[55]
S100A7	NR	92	NR	Mean AAbs level was significantly higher in ovarian carcinoma patients as compared to that healthy controls. Potential diagnostic value.	[2]

NR, not recorded.
